# First Evidence of Palytoxin and 42-Hydroxy-palytoxin in the Marine Cyanobacterium *Trichodesmium*

**DOI:** 10.3390/md9040543

**Published:** 2011-03-31

**Authors:** Anne Sophie Kerbrat, Zouher Amzil, Ralph Pawlowiez, Stjepko Golubic, Manoella Sibat, Helene Taiana Darius, Mireille Chinain, Dominique Laurent

**Affiliations:** 1 Toulouse University, UPS, UMR152 UPS-IRD (PHARMA-DEV), 118, route de Narbonne, F-31062 Toulouse cedex 9, France; E-Mail: kerbrat@ael-environnement.nc; 2 Research Institute for the Development (IRD), UMR152, 98848 Noumea, New Caledonia; 3 Laboratory of Phycotoxins, IFREMER, Rue de l’Ile d’Yeu, BP21105, F-44311 Nantes cedex 3, France; E-Mails: Zouher.Amzil@ifremer.fr (Z.A.); Manoella.Sibat@ifremer.fr (M.S.); 4 Laboratory of toxic micro-algae (LMT), Louis Malarde Institute (ILM), BP30, 98713 Papeete, Tahiti, French Polynesia; E-Mails: rpawlowiez@ilm.pf (R.P.); tdarius@ilm.pf (H.T.D.); mchinain@ilm.pf (M.C.); 5 Biological Science Center, Boston University, 5 Cummington Street, Boston, MA 02215, USA; E-Mail: golubic@bu.edu; 6 Research Institute for the Development (IRD), UMR152, 98713 Papeete, Tahiti, French Polynesia

**Keywords:** cyanobacteria, *Trichodesmium*, palytoxin, 42-hydroxy-palytoxin, clupeotoxism

## Abstract

Marine pelagic diazotrophic cyanobacteria of the genus *Trichodesmium* (Oscillatoriales) are widespread throughout the tropics and subtropics, and are particularly common in the waters of New Caledonia. Blooms of *Trichodesmium* are suspected to be a potential source of toxins in the ciguatera food chain and were previously reported to contain several types of paralyzing toxins. The toxicity of water-soluble extracts of *Trichodesmium* spp. were analyzed by mouse bioassay and Neuroblastoma assay and their toxic compounds characterized using liquid chromatography coupled with tandem mass spectrometry techniques. Here, we report the first identification of palytoxin and one of its derivatives, 42-hydroxy-palytoxin, in field samples of *Trichodesmium* collected in the New Caledonian lagoon. The possible role played by *Trichodesmium* blooms in the development of clupeotoxism, this human intoxication following the ingestion of plankton-eating fish and classically associated with *Ostreopsis* blooms, is also discussed.

## 1. Introduction

*Trichodesmium* spp. are marine pelagic cyanobacteria belonging to the order Oscillatoriales. These filamentous, non-heterocystous cyanobacteria are known for their ability to fix atmospheric dinitrogen [1–3]. They are characterized by trichomes (linear arrangements of about 100–200 cells) that form colonies and occur in extensive floating blooms also called “sea sawdust” by the sailors. *Trichodesmium* blooms are widely distributed in oligotrophic regions of the oceans throughout the tropics and subtropics [1,4,5].

Despite a number of surveys dedicated to the ecological aspects of *Trichodesmium* spp. and their importance for the coral reef ecosystems [1,3,6,7], their toxicity remain sparsely documented [3,8]. The stochastic nature of the blooms (and the difficulties inherent in establishing laboratory cultures) has greatly hampered toxicological studies [9,10]. In 1991, Hawser [11] reported the death of oysters following *Trichodesmium* blooms. The toxicity of these cyanobacteria was tested on various species of zooplankton and mortality of certain crustaceans (brine shrimp and two species of copepods) was demonstrated. However, grazers (*Macrosetella gracilis* and *Miracia efferata*) that are known to feed on *Trichodesmium* were not affected. No information was provided on the nature of the toxins involved [11–13]. Based on chemical analysis studies, Hahn and Capra [14] were the first to hypothesize that *Trichodesmium erythraeum* could be a potential source of toxin in ciguatera, a typical foodborne intoxication in the tropics due to the ingestion of fish contaminated with ciguatoxins (CTXs) [15–17]. The compounds extracted from *T. erythraeum* and from samples of molluscs, collected during, and shortly after, these *Trichodesmium* blooms, were positive for CTXs-like toxins [14]. In 1993, Endean *et al.* [18] demonstrated that the toxin profiles of the lipid- and water-soluble extracts from *T. erythraeum* were similar to those of corresponding fractions extracted from the flesh extracts of the pelagic carnivore *Scomberomorus commerson*, a fish often implicated in ciguatera. Chromatographic elutions of water-soluble and lipid-soluble fractions from both *Trichodesmium* and *Scomberomorus* samples further showed the presence of an alkaloid, in addition to a peptide and CTXs-like compounds. These observations, as well as recent studies by Kerbrat *et al.* [19], conducted mainly on *T. erythraeum* blooms in the New Caledonia lagoon, tend to indicate that *Trichodesmium* spp. could be the source of some of the toxins carried by ciguateric fish, and may contribute to the ciguatera syndrome. Recently, Ramos *et al.* [20] detected the presence of microcystin-LR in *T. erythraeum* by chromatographic analysis. Moreover, Proença *et al.* [21] analyzed the contents of analogues of microcystin, cylindrospermopsin and saxitoxin in *Trichodesmium* blooms off the Brazilian coasts. Saxitoxin analogues and microcystins were present at low concentrations in all samples, but the authors concluded that these toxins do not represent a potential harm to human health by primary contact. The only reported harmful effect of *Trichodesmium* to humans refers to the “Tamandare fever” on the coast of Tamandare, Brazil [22]. The possible involvement of marine benthic cyanobacteria in ciguatera outbreaks in New Caledonia has recently been documented by Laurent *et al.* [23]. The incriminated cyanobacterium *Hydrocoleum lyngbyaceum* was found phylogenetically very close to the species of *Trichodesmium* [24,25]. The presence of homoanatoxin-a, a derivative of anatoxin-a, in mats of *H. lyngbyaceum*, as well as in giant clams (*Tridacna* spp.) collected in the surroundings of contaminated area, has been recently reported by Méjean *et al.* [26]. Both these neurotoxins are well known in freshwater cyanobacteria involved in dog poisonings in France, New Zealand and Scotland [27,28].

**Figure 1.**
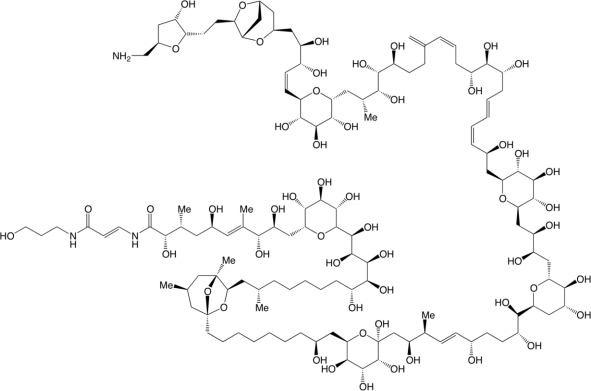
Structure of palytoxin from Riobó [38].

Originally, palytoxin (PLTX) (Figure 1) and 42-OH-palytoxin (42-OH-PLTX) were isolated from the zoanthid anemone *Palythoa* sp. [29,30]. PLTX and analogues like ovatoxins, ostreocins, ostreotoxins, mascarenotoxins constitute the family of PLTXs [31]. PLTXs were evidenced in marine organisms ranging from dinoflagellates (*Ostreopsis*) to fishes [32–35] but, to our knowledge, never in cyanobacteria. PLTX is one of the largest nonpolymeric natural molecules with a molecular weight of 2680 Da and one of the most potent non-protein compounds known to date, exhibiting high toxicity in mammals with intravenous LD_50_ ranging between 10 and 100 ng/kg [31–33]. One of the main actions of PLTX is to bind to the Na, K-ATPase, converting the pump into an ion channel and causing a K^+^ efflux, an Na^+^ influx and membrane depolarization [32]. The osmotic imbalance that results from this flux of ions can be compared to CTX mechanisms and could explain why PLTX has often been implicated in ciguatera [36,37].

PLTX is also likely to play a role in clupeotoxism, a marine poisoning resulting from the ingestion of plankton-eating fish such as herrings and sardines (Clupeidae), anchovies (Engaulidae) or mullets (Mugillidae) in tropical regions [37,39]. This has been postulated after the detection of PLTX and analogues in the remains of fish instigating serious human intoxications [36,40,41]. Symptoms appear abruptly: metallic taste, digestive disorders, generalized paralysis, tachycardia, convulsions and acute respiratory distress. Their variety and intensity depend upon the route of exposure which occurred through the consumption of PLTX-contaminated organisms and through dermal, ocular and inhalation routes [42]. Although rare, this poisoning can be fatal [37]. Clupeotoxism is classically associated with blooms of the benthic dinoflagellate *Ostreopsis*, most notably with the species *O. siamensis* and *O. mascarenensis* known as sources of PLTX [43,44] whereas two other species, *O. lenticularis* and *O. ovata*, are potentially progenitors of PLTX analogues: ostreotoxins and ovatoxin-a, respectively [45].

Detection and quantification of PLTX in biological samples can be conducted by biological and analytical means, but there is currently no officially approved method [38,46]. Here, we used two biological methods (mouse bioassay and Neuroblastoma cell-based assay) and one analytical method (LC-MS/MS) to detect this toxin in marine cyanobacteria.

The present contribution provides the first evidence of the production of PLTX and one of its analogues, 42-OH-PLTX by *Trichodesmium* in tropical and subtropical waters. The potential role played by *Trichodesmium* blooms in clupeotoxicity, via the ingestion of the trichomes of this pelagic cyanobacterium by plankton-eating fish is also discussed.

## 2. Materials and Methods

### 2.1. Materials

All reagents and chemicals were obtained from Sigma-Aldrich (St. Louis, MO, USA) unless otherwise stated. Solvents used for extraction and purification were of analytical grade and were purchased from Prolabo (Paris, France). For chromatographic techniques, methanol and acetonitrile were HPLC grade, obtained from J.T. Baker (Deventer, The Netherlands). Water was deionised and purified to 18.2 MΩ quality through a MilliQ water purification system (Purelab Elga, UK). Standard solution of PLTX was purchased from Wako chemicals GmbH (Neuss, Germany). In addition to PLTX, this solution contains traces of ovatoxin-a and 42-OH-PLTX.

### 2.2. Sampling Sites and Collection of Cyanobacteria

**Figure 2.**
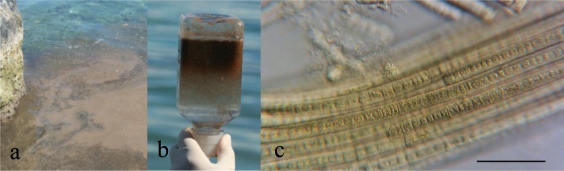
A bloom of *Trichodesmium erythraeum* Ehrenberg: (**a**) Field view of wind-blown concentration of colonies; (**b**) Concentration of trichomes using their buoyancy properties; (**c**) *Trichodesmium* trichomes in bundles oriented in parallel (scale bar = 50 μm).

*Trichodesmium* sampling took place in the southern lagoon of New Caledonia, primarily during the southern summer (September to March), as soon as the blooms were reported (Table 1). Massive blooms were subject to drifts by wind and concentrated especially in bays (Figure 2a). Samples were collected on the surface and sub-surface (0–1 m). Two sampling techniques were used, depending on trichome concentrations: manually with a 35 μm phytoplankton net or with a gentle suction using a vacuum pump. All samples were handled delicately to avoid cell lysis leading to toxin release. The buoyancy of cyanobacteria conferred by intracellular gas vesicles separates them from debris and other organisms (Figure 2b). The trichomes were separated from remaining macroalgae, phanerogams and debris and further concentrated by reversing the sampling bottles. Concentrated samples were frozen and kept freeze-dried until extracted and tested for their toxicity. Subsamples from each batch were fixed in 5% formaldehyde solution in Millipore^®^-filtered sea-water (0.45 μm) for morphological identification purposes.

### 2.3. Taxonomic Identification of Cyanobacterial Samples

Samples collected from various occurrences of *Trichodesmium* blooms and different locations are presented in Table 1. *T. erythraeum* is known to be prevalent in the New Caledonian lagoon [7]. *T. erythraeum* forms typically spindle-shaped colonies composed of trichomes oriented in parallel (Figure 2c) [47]. Cells are shorter (5.4–11 μm) than wide (7–11 μm). The end cells are often capped by a calyptra. Although associated with a variety of organisms, including hydrozoans, copepods, diatoms, dinoflagellates, fungi and other protists and bacteria, *Trichodesmium* species are usually the major component of the blooms.

### 2.4. Extraction

Freeze dried pellets of cyanobacterial samples were extracted using solvent partition. Briefly, pellets (≈100 g) were extracted three times with methanol (1 L) with each time one hour of ultra-sonication and by agitation overnight. This extract was subsequently filtered and dried under vacuum, and the residue was partitioned between 60% aqueous methanol (500 mL) and diethyl ether (250 mL). The water-soluble fraction was saved and dried under vacuum. Data concerning the respective extraction yields are summarized in Table 1.

### 2.5. Mouse Bioassay

The mouse bioassays (MBA) were based on careful observation of the symptoms displayed by the animals following injection of toxic extracts. MBA were performed on aqueous methanolic extracts using 20 g ± 2 g mice (OF1, Iffa-Credo, L’Arbresle, France) of either sex. All tested animals were treated under conditions, which meet the ethical standards defined by the European Community Council Directive of November 24, 1986 (86/609/EEC). The animals were allowed food and water *ad libitum*.

The dried extracts were dissolved in 300 μL of phosphate buffer saline (PBS, pH 7.2) containing 0.1% Tween 80, prior to administration via intraperitoneal (i.p.) injection. The tested doses varied from 0.5 to 5.0 mg of extract/g of mouse body weight (*n* ≥ 2; 3 different concentrations depending on the extract). In total, six animals were used per extract. Control animals received 300 μL of vehicle (*n* = 2). Animal behavioral changes were observed over a period of approximately 48 h.

### 2.6. Neuroblastoma Cell-Based Assays (CBA)

The Neuroblastoma cell-based assays (CBA) was performed to quantify the cytotoxic effect of water-soluble extract following the method previously described by Ledreux *et al.* [48] with some modifications described below. The Neuroblastoma cells (Neuro-2a) were obtained from the American Type Culture Collection (ATCC CCL 131). Neuro-2a cells were maintained in RPMI-1640 medium supplemented with 1 mM sodium pyruvate, 2.5 μg/mL amphotericin B, 50 units/mL penicillin G, 50 μg/mL streptomycin sulfate, 1% glutaMAX™-I, and 10% FBS (fetal bovine serum), at 37 °C in a humidified 5% CO_2_ atmosphere. Briefly, Neuro-2a were harvested with a trypsin-EDTA solution and 50,000 cells in a 5% FBS RPMI-1640 supplemented medium were seeded into each well of a 96-well microtiter plate, and incubated for 24 h at 37 °C.

#### 2.6.1. Evaluation of Cytotoxic Effects of PLTX

The incubation step in a 96-well microtiter plate was followed by a pre-treatment for 2 h with a control solution or ouabain solution at 100, 250 or 500 μM. Different pre-treatment times (0, 1 and 2 h) with ouabain 500 μM were also tested. The final PLTX concentrations ranged from 1.8 × 10^−15^ to 1.8 × 10^−8^ M. At least 3 replicates per dilution were tested and for each microplate, 6 wells were processed as untreated controls and 6 wells as ouabain control. After a 20–22 h incubation period at 37 °C, cell viability was assessed using the quantitative colorimetric 3-(4,5-dimethylthiazol-2-yl)-2,5-diphenyl tetrazolium bromide (MTT) assay, a method previously described by Mossman [49]. Medium was removed, and 60 μL of medium containing 0.83 mg/mL of MTT were added to each well. The plates were incubated for 1 h at 37 °C. Finally, the MTT was discarded and 100 μL dimethyl sulfoxide added to each well to dissolve the formazan. The absorbance was read at 570 nm on a plate reader (Imark microplate reader, BioRad). Compared to the absorbance of cells alone (100% of viability), the results were expressed as the percentage of viability. Data were fitted to a sigmoid curve with variable slope using GraphPad Prism version 4. EC_50_ values (concentration of toxin or extract that reduces by half the cell survival) were determined for samples that showed a sigmoidal curve. R^2^ values, not shown here, were found higher than 0.97.

#### 2.6.2. Evaluation of Cytotoxic Effects of the Extracts

The incubation step was followed by a pre-treatment for 2 h with a control solution, or with an ouabain solution at a final concentration of 500 μM. Different dilutions of the toxic extracts were then added to each well, at a final concentration ranging from 178.5 to 4464 μg/mL followed by 20–22 h incubation and analyzed using MTT assay.

#### 2.6.3. Evaluation of Cytotoxic Effects of Extracts Spiked with PLTX

To evaluate the effectiveness and the specificity of the CBA for PLTXs and the matrix effect on Neuroblastoma cells, a non-toxic extract was spiked on pure PLTX. The sample No. 8 (Tricho Lifou C02) was spiked to obtain a final concentration of 1.7 μg PLTX/g of extract corresponding to the concentration estimated in the most toxic extract (No. 1, Tricho 5îles) by chromatographic analyses. Different dilutions of the spiked extract were then added to each well: (i) at a final extract concentration ranging from 178.5 to 4,464 μg/mL, or (ii) at a final PLTX concentration ranging from 6.4 × 10^−9^ to 1.6 × 10^−7^ M with pre-treatment for 2 h with ouabain (500 μM), followed by MTT assay.

### 2.7. LC-MS/MS Analysis

Liquid Chromatography coupled with tandem mass spectrometry (LC-MS/MS) analyses was performed with aqueous methanol 80% extracts. An aliquot (300 μL) was filtered through a 0.2 μm Whatman^®^ Vectaspin filter. Five microliters of the filtrate were injected for analyses by LC-MS/MS.

PLTXs analysis were carried out using an LC system (HP 1200, Agilent) coupled to a hybrid triple quadrupole/ion trap mass spectrometer (API-4000Qtrap, PE/SCIEX) equipped with a turbo spray^®^ interface, according to modified Ciminiello method [45]. A C18 Gemini column (5 μm, 150 mm × 2.0 mm, Phenomenex) was employed at 20 °C and eluted at 200 μL/min. Eluent A was water and eluent B was 95% acetonitrile/water, both eluents containing 2 mM ammonium formate and 50 mM formic acid. The gradient of B was raised from 20 to 100% over 10 min and held for a further 4 min before returning to initial conditions. The instrument control, data processing and analysis were conducted using Analyst software.

Mass spectrometry detection was performed in positive mode and optimized from a PLTX standard solution using Selected Reaction Monitoring (SRM) (Table 2). The SRM experiments were established by using the following source settings: curtain gas set at 30, ion spray at 5500 V, a turbogas temperature of 450 °C, gas 1 and 2 set at 50 (arbitrary units) and an entrance potential of 10 V. The collision energy was applied at 45 eV for doubly charged ions [M + 2H]^2+^, [M + 2H − H_2_O]^2+^ and at 33 eV for triply charged ions [M + 3H]^3+^ to give the characteristic product ion at 327.

## 3. Results

### 3.1. MBA Toxicity Data

The five water-soluble extracts of *Trichodesmium* injected (No. 1, 4, 5, 6 and 8) were found toxic. Symptoms in mice included reduced activity and responsiveness, frequent convulsive spasms, respiratory difficulty and partial paralysis, which quickly advanced to total paralysis. No salivation or lacrimation was observed. The i.p. injection of 2.5 mg of water-soluble extracts/g of mouse body weight (corresponding to *ca.* 12 mg of freeze-dried pellets of cyanobacteria/g of mouse body weight) killed all mice within 5 min. Below this concentration, a complete recovery of mice was observed accompanied with a transient comatose phase lasting from 40 min to 2 h. The extracts No. 2, 3, and 7 were not injected in mice.

### 3.2. CBA Cytotoxicity Data

#### 3.2.1. Effect of PLTX

**Figure 3.**
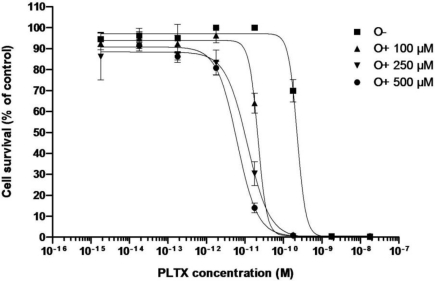
Effect of PLTX alone on the viability of Neuro-2a cells, and with pre-incubation with 100, 250 or 500 μM ouabain for 2 h before adding PLTX.

**Figure 4.**
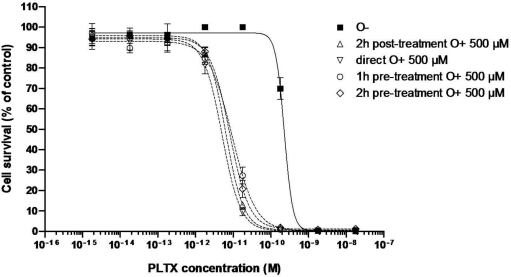
Effect of PLTX alone on the viability of Neuro-2a cells, and with pre-incubation of PLTX for 2 h before adding 500 μM ouabain, with 500 μM ouabain administered simultaneously, with pre-incubation of 500 μM ouabain for 1 h or 2 h before adding PLTX.

**Figure 5.**
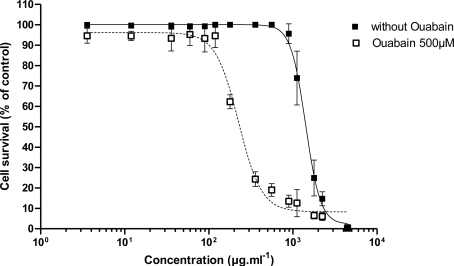
Effect of *Trichodesmium* extract No. 1, “5îles”, alone on the viability of Neuro-2a cells and with pre-incubation with 500 μM ouabain for 2 h before adding extract.

EC_50_ values for the dose-response curves obtained for the direct effect of PLTX or after pre-incubating the Neuro-2a cells with 100, 250 or 500 μM ouabain (O) and for different pre-incubation times (0, 1 or 2 h) are presented in Table 3. The dose-response curves with different ouabain concentration are presented in Figure 3 while the dose-response curves with different pre-incubation time are presented in Figure 4. Following a pretreatment to ouabain (2 h), Neuro-2a cells were sensitized in a positive dose-dependent manner to the action of PLTX (Figure 3). The action of ouabain on the sensitivity of Neuro-2a to PLTX did not depend on its administration time (Figure 5). In all cases, ouabain sensitized the Neuro-2a cells.

#### 3.2.2. Effect of *Trichodesmium* Extracts

EC_50_ values for the dose-response curves obtained for the effect of *Trichodesmium* extracts alone or after pre-incubating the Neuro-2a cells for 2 h with 500 μM ouabain were presented in Table 2. The effect of *Trichodesmium* extract No. 1 from “5îles” on the viability of Neuro-2a cells, without or with pre-incubation with 500 μM ouabain for 2 h before adding extract is illustrated in Figure 5. As in the experiments with PLTX, the sensitivity of the Neuro-2a cells to the *Trichodesmium* extract No. 1 has increased with pre-incubation with 500 μM ouabain for 2 h (Figure 5).

#### 3.2.3. Effect of *Trichodesmium* Non-Toxic Extracts Spiked with PLTX

**Figure 6.**
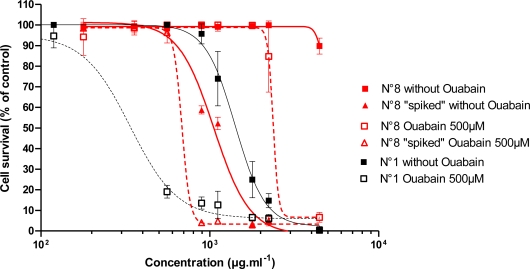
Effect of *Trichodesmium* extract (No. 8) from Lifou on the viability of Neuro-2a cells alone or spiked with an equivalent of 1.7 μg PLTX/g of extract, and with pre-incubation with 500 μM ouabain for 2 h before adding extract. Toxicity of *Trichodesmium* extract No. 1 from 5îles was compared with the toxicity of *Trichodesmium* extract No. 8 and with pre-incubation with ouabain.

When the non-cytotoxic extract (Tricho Lifou C02, No. 8) was spiked with a PLTX dose equivalent at 1.7 μg of PLTX/g of extract (PLTX content of the most toxic *Trichodesmium* extract No. 1, Tricho “5îles”), this atoxic extract became toxic with an EC_50_ of 1058 μg/mL as opposed to 1337 μg/mL for extract No. 1. With 2 h of pre-incubation, EC_50_ decreased from 2054 to 683 μg/mL as opposed to 114 μg/mL for non-spiked extract No. 1 (Figure 6).

Without pre-treatment with ouabain, the cytotoxicity of the extract No. 8 spiked with PLTX is slightly stronger than that of the natural toxic extract No. 1. With pre-incubation with ouabain, we observed the inverse phenomenon: a stronger cytotoxic potential of the non-spiked extract than that of the spiked extract.

### 3.3. LC-MS/MS Analysis

**Figure 7.**
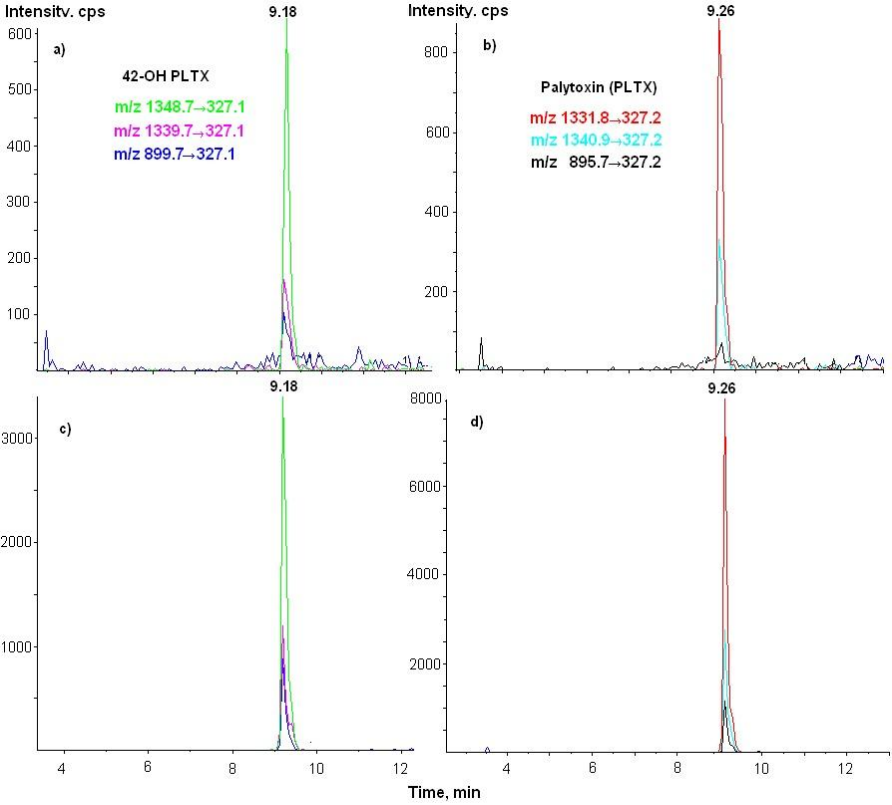
LC-MS/MS chromatograms of 42-OH-PLTX (**a**) and PLTX (**b**) in a sample of *Trichodesmium* spp. Standards of 42-OH-PLTX (**c)** and PLTX (**d**) were purchased from Wako.

Several toxins were submitted to LC-MS/MS analysis: PLTX, 42-OH-PLTX, ovatoxin-A (analogue of PLTX isolated from *Ostreopsis* cf. *ovata*), ostreocin-D (analogue of PLTX isolated from *O. siamensis*), mascarenotoxins A and B (analogue of PLTX isolated from *O. mascarensis*). Eight water extracts were tested: among all the toxins screened, only PLTX and 42-OH PLTX were detected in four samples of *Trichodesmium* (No. 1, 4, 5, 6) (Figure 7, Table 4). No toxins were detected in the samples No. 2, 3, 7, 8 (Limit of Detection = 0.01 μg of PLTX/g of extract). In the extract containing PLTX and 42-OH-PLTX, the concentration of these combined toxins varies from 1.08 to 1.70 μg/g of extract (0.28 to 1.10 μg/g eqv. of dried cyanobacteria) (Table 4).

## 4. Discussion

The first evidence of the production of PLTX and one of its analogues, 42-hydroxy-palytoxin (42-OH-PLTX), by *Trichodesmium* cyanobacteria in New Caledonian waters is demonstrated by CBA and LC-MS/MS.

In our experiments, Neuro-2a cells seemed to be more sensitive to PLTX standards and to both cytotoxic and atoxic extracts after a pre-treatment with ouabain, whereas according to Ledreux *et al.* [48], the presence of ouabain, in counteracting the effects of PLTX on Na^+^/K^+^ ATPase, should inhibit partially the cytotoxicity of PLTX or PLTX-contaminated extracts. This PLTX dose-dependent decrease in viability was also specifically inhibited by ouabain in the case of BE (2)-M17 Neuroblastoma cells [50]. Ouabain also showed inhibition of the lysis of sheep erythrocytes by PLTX [51].

Studying the suitability of the Neuro-2a cell line for the detection of PLTX and analogs, Ledreux *et al.* [48] found an EC_50_ value of 42.9 pM for a direct cytolitic effect of PLTX, and an EC_50_ value of 290.7 pM for a specific effect of PLTX when ouabain was used as a competitor and pre-added 2 h before, at the concentration of 500 μM. For our part, we found an EC_50_ of 170 ± 60 pM for direct effect of PLTX and 6.0 ± 2.2 pM for the specific effect, respectively, in the same conditions, *i.e.*, ouabain concentration and pre-incubation time (Table 3). The CBA method was largely based on Ledreux’s method, with the exception that after 24 h incubation time, the medium was not removed and replaced by MWS (Medium without Serum) for economic reasons, and that the final working volume was 112 μL. We also tried Ledreux’s method entirely (data not shown), and we made the same observations as previously found, that Neuro-2a with 2 h ouabain 500 μM pre-treatment were more sensitive to PLTX than cells without pre-treatment.

To explore the observed difference in the specific effect of PLTX after pre-incubation of Neuro-2a cells with ouabain, compared to literature, different sets of experiments were performed with respect to ouabain concentration and pre-incubation time (Table 3). As a result, the highest sensitivity of the Neuro-2a cells was observed when ouabain was added prior to PLTX. This sensitivity was dose-dependent (from 100 to 500 μM ouabain) (Figure 3) and was not dependent of pre-incubation time (Figure 4).

Cañete and Diogene [52] obtained in 24 h growth and 24 h exposure conditions, a dose-response curve with an EC_50_ of 100 pM without ouabain/veratridine (O/V) and 40 pM with O/V added simultaneously with the toxins. Ouabain and veratridine treatment affects the ionic cell equilibrium; therefore, cells treated with O/V were more sensitive to PLTX than the untreated cells. The synergistic effect of PLTX and ouabain was observed (EC_50_ = 6.3 ± 4.7 pM) when these compounds were both added simultaneously to the Neuro-2a cells [48]. Our results were very similar with an EC_50_ of 5.08 pM or 6.37 with a post-treatment of 500 μM ouabain, respectively, showing that our ouabain sample is active (Table 3).

The mouse bioassay revealed a global toxicity of the five tested extracts with paralyzing effect. No difference was noted in the activity and toxic potency of *T. erythraeum* blooms collected from different locations.

However, the bioassay with Neuroblastoma cells seems to show the potential cytotoxicity of extracts even if the results do not fully correlate with the presence or the absence of PLTX detected by LC-MS/MS. Only one of the eight extracts, the bloom collected in Nouméa (No. 7), was found cytotoxic while PLTX was not detected by LC-MS/MS. It is possible that the toxicity in mice and the cytotoxicity observed on Neuro-2a cells is due to other paralyzing toxins or cytotoxins. However, all these samples were analyzed by LC-MS/MS for their content in cyanotoxins (anatoxin-a, homoanatoxin-a, cylindrospermopsin, nodularin-R, microcystins), paralyzing shellfish toxins (STX, NEO-STX, GTX1 to GTX6, C1 to C4, dcSTX, dcNEO, dcGTX1 toDCGTX4), lipophilic toxins (okadaic acid, dinophysistoxins, pectenotoxins, azaspiracids, yessotoxins) and fast-acting toxins (spirolides and gymnodimines) but none of these was detected. In addition, cyanobacteria, proliferating in marine environments, are an important source of structurally diverse bioactive secondary metabolites. Some of these compounds show a strong cytotoxicity (lyngbyatoxins, lyngbyabellins, aplysiatoxins, dolastatins, curacin, aurilide, antillatoxin, kalkitoxin, jamaïcamide) which could interfere if these compounds were present.

According to the results of LC-MS/MS analysis, four extracts from eight *Trichodesmium* blooms appeared to contain PLTX and its congener, 42-OH-PLTX. This presence of PLTX depends neither on the location nor the collection season. The four non-toxic extracts may come from blooms composed either of non-toxigenic strains, or from blooms that were harvested at a physiological level close to senescence. Indeed, these non-toxic extracts came from blooms of weak orange color with white streaks, which may have already lost some of their pigments; the lysis of the cells may have already begun, causing the release of toxins in the environment.

The concentrations of PLTX in the toxic samples, detected by CBA or by LC-MS/MS, are relatively low, the highest being 1.70 μg/g of aqueous extract or 1.1 μg equivalent of total PLTX/g of freeze-dried material (Table 4). The maximum levels of PLTX in shellfish are not regulated, but a health value was proposed by the European Food Safety Authority (EFSA) in 2009 as 0.03 μg/g of flesh [53]. Given this level, *Trichodesmium* blooms could lead to a risk for human health by bathing but also by consumption of shellfish or fish, which, in addition, could absorb and bioaccumulate these toxins.

*Trichodesmium* colonies constitute a living habitat for a variety of small marine organisms [54,55]. They are also consumed by certain invertebrate species showing tolerance to the concentrated toxins, like the pelagic copepods, *Macrosetella gracilis* and *Miracia efferata* [8,13,54]. Accordingly, the toxins produced by these cyanobacteria have the potential to enter the food chain. Mullets (*Mugilidae*), for instance, are known to graze on *Trichodesmium* as reported by local fishermen, which also reported cases of ciguatera-like intoxications following the ingestion of this fish. During the course of this study, we could personally observe schools of mullets grazing on the large bloom of *Trichodesmium* harvested in Lifou in November 2008 and 2009. In addition, Endean *et al.* [18] demonstrated that the toxins produced by *T. erythraeum* were indistinguishable from those present in the flesh of the narrow-barred Spanish mackerel *Scomberomorus commerson*, frequently implicated in Ciguatera Fish Poisoning (CFP) outbreaks. Wachi *et al.* [51] using a hemolysis neutralization assay with both ouabain and an anti-PLTX antibody showed that a moderate percentage of gut extracts from herbivorous reef fish, and flesh extracts from carnivorous species did exhibit PLTX-like-activities. Then, combining mouse bioassay and hemolysis neutralization assay, Wachi and Hokama [56] concluded that several toxins, PLTX-like compounds and probably CTX-like compounds, appear to be present in herbivorous and carnivorous Hawaiian reef fishes. The presence of PLTX in flesh of some carnivorous species is surprising given the water soluble nature of PLTX [37].

Recently, our studies supported the hypothesis of the presence of CTX-like compounds in *Trichodesmium* blooms [19]. The combination of these toxins with paralyzing toxins such as PLTX, may lead to poisoning described by inhabitants of New Caledonia after consumption of mullets. As was the case for saxitoxins, CTX-like compounds and PLTX are two types of toxins that were thought to be of marine dinoflagellate origin, but which may also be produced by a prokaryote.

In conclusion, *Trichodesmium* blooms are an esthetically unpleasant nuisance, which could become a health hazard to swimmers, fishermen and researchers repeatedly exposed during collection. Moreover, by bioaccumulation of PLTX and congeners, they may cause a danger to consumers of planktivorous fish that have ingested trichomes of cyanobacteria. To confirm this hypothesis and the involvement of *Trichodesmium* in the clupeotoxism, further studies are required, including analyses of PLTX content of planktivorous fish in contact with a bloom.

## Figures and Tables

**Table 1.** *Trichodesmium* collections: date, location, identification and yield extraction.

**No.**	**Date**	**Reference**	**Location**	**Latitude**	**Longitude**	**Water-soluble fraction % of dried material**

1	2007-03-01	Tricho 5îles	5îles	−22.771900	166.800995	64.3
2	2007-09-24	Tricho BD 2007	Baie des citrons	−22.297600	166.438004	51.6
3	2008-02-08	Tricho BD 2008	Baie des citrons	−22.295700	166.436005	68.8
4	2008-02-18	Tricho Dumbéa	Passe de Dumbéa	−22.349501	166.274994	26.2
5	2008-02-18	Tricho Ricaudy	Récif Ricaudy	−22.306900	166.460210	21.5
6	2008-11-04	Tricho Lifou C01	Lifou–Hunëtë	−20.767310	167.093006	52.5
7	2009-02-01	Tricho Nouméa 3	Passe de Dumbéa	−22.349501	166.274994	49.7
8	2009-11-18	Tricho Lifou C02	Lifou–Hunëtë	−20.767310	167.093006	55.4

**Table 2.** SRM parameters setting used for PLTXs-like detection.

**Toxins**	**Transitions *m/z***	**Declustering potential (V)**	**Cell exit potential (V)**	**Dwell time (ms)**
**PLTX**	1340→327	26	18	250
	1332→327	26	18	250
	896→327	61	8	250
**Ovatoxin-a**	1324→327	26	18	250
	1315→327	26	18	250
	889→327	61	8	250
**42-OH-PLTX**	1348.7→327	26	18	250
	1339.7→327	26	18	250
	899.7→327	61	8	250

**Table 3.** EC_50_ values of the dose-response curves obtained for the PLTX with or without ouabain pre-incubation *.

**Conditions**	**Without O**	**Pre PLTX**	**O**	**Pre O**			
O (μM) Pre-incubation time (h)	0	500 2	500 0	500 1	500 2	250 2	100 2
C_50_ (pM)	170 ± 60 (*n* = 3)	6.37	5.08	9.41	6.0 ± 2.2 (*n* = 3)	11.65	21.74

* Neuro-2a cells with 100, 250 and 500 μM ouabain (O) with different preincubation times (0, 1 and 2 h). R^2^ values showed always good fit. Each point represents at least the mean of 3 well values. For some conditions (without O and PreO 500, 2 h), data represent the mean ±SD of 3 separate experiments.

**Table 4.** Results of Neuro-2a cells cytotoxicity (CBA) and LC-MS/MS analysis *.

**No.**	**Reference**	**CBA**		**LC-MS/MS**			
		**preO−**	**preO+**	**PLTX**	**42-OH-PLTX**	**Total PLTX eqv.**	
		**EC_50_** **μg/mL**		**μg/g extract**		**μg/g extract**	**μg/g eqv. dried material**

1	Tricho 5îles	1337 ± 126	113.8 ± 110.8	0.82	0.87	1.70	1.10
2	Tricho BD 2007	>LOQ	2261	<LOD	<LOD	<LOD	<LOD
3	Tricho BD 2008	>LOQ	1138	<LOD	<LOD	<LOD	<LOD
4	Tricho Dumbéa	1324/1066	494/158	0.57	0.52	1.08	0.28
5	Tricho Ricaudy	927	NA	0.89	0.64	1.53	0.33
6	Tricho Lifou C01	1214	397	0.86	0.59	1.45	0.76
7	Tricho Nouméa 3	1212	91	<LOD	<LOD	<LOD	<LOD
8	Tricho Lifou C02	>LOQ	2054 ± 277	<LOD	<LOD	<LOD	<LOD

* preO−: Without ouabain pre-treatment; preO+: With ouabain pre-incubation; LOQ (Limit of Quantification): 4464 μg/mL for CBA; LOD (Limit of Detection): 0.01 μg/g for LC-MS/MS; NA: Not Available.
